# Some aggregation operators of neutrosophic Z-numbers and their multicriteria decision making method

**DOI:** 10.1007/s40747-020-00204-w

**Published:** 2020-11-01

**Authors:** Shigui Du, Jun Ye, Rui Yong, Fangwei Zhang

**Affiliations:** 1 Institute of Rock Mechanics, Ningbo University, Ningbo, 315211 People’s Republic of China; 2 Department of Civil Engineering, Shaoxing University, Shaoxing, 312000 People’s Republic of China

**Keywords:** Neutrosophic Z-number set, Neutrosophic Z-number, Neutrosophic Z-number weighted arithmetic averaging operator, Neutrosophic Z-number weighted geometric averaging operator, Multicriteria decision making

## Abstract

As the generalization of the classical fuzzy number, the concept of Z-number introduced by Zadeh indicates more ability to depict the human knowledge and judgments of both restraint and reliability as an order pair of fuzzy numbers. In indeterminacy and inconsistent environment, a neutrosophic set is described by the truth, falsity, and indeterminacy degrees, but they lack measures related to reliability. To describe the hybrid information of combining the truth, falsity and indeterminacy degrees with their corresponding reliability degrees, this paper first proposes the concept of a neutrosophic Z-number (NZN) set, which is a new framework of neutrosophic values combined with the neutrosophic measures of reliability, as the generalization of the Z-number and the neutrosophic set. Then, we define the operations of neutrosophic Z-numbers (NZNs) and a score function for ranking NZNs. Next, we present NZN weighted arithmetic averaging (NZNWAA) and NZN weighted geometric averaging (NZNWGA) operators to aggregate NZN information and investigate their properties. Regarding the NZNWAA and NZNWGA operators and the score function, a multicriteria decision making (MDM) approach is developed in the NZN environment. Finally, an illustrative example about the selection problem of business partners is given to demonstrate the applicability and effectiveness of the developed MDM approach in NZN setting.

## Introduction

It is known that fuzzy sets proposed by Zadeh [1] play an essential role in the current scientific and technical applications [2–7]. In 2011, Zadeh [8] further introduced the concept of Z-numbers to describe the restraint and reliability of the evaluation by an order pair of fuzzy numbers in uncertain situations. Compared with the classical fuzzy number, it is a more generalized notion closely related to reliability. Hence, the Z-number implies more ability to describe the human knowledge and judgments by an order pair of fuzzy numbers corresponding to the restriction and reliability. Since then, it has obtained a lot of attentions. Some researchers presented theoretical studies of Z-numbers, like Z*-numbers [9], arithmetic operations of discrete and continuous Z-numbers [10, 11], modeling of Z-number [12], approximate reasoning of Z-numbers [13], functions based on a Z-number set [14], total utility of Z-numbers [15] and so on; while other researchers developed some applications of Z-numbers, such as Z-evaluations [16], sensor data fusion using Z-numbers [17], decision making approaches with Z-numbers [18–24], Z-numbers-based stable strategies analysis in evolutionary game [25], Z-numbers-based medicine selection of the patients with mild symptoms of the COVID-19 [26], Z-numbers-based environmental assessment under uncertainty [27] and so on.

In indeterminate and inconsistent environment, neutrosophic sets [28, 29] are described independently by the truth, falsity, and indeterminacy membership degrees, but the aforementioned Z-numbers cannot depict them. Then, neutrosophic sets have been applied in various areas, such as image processing [30], decision making [31–34], medical diagnosis [35–37], and mechanical fault diagnosis [38]. However, the truth, falsity, and indeterminacy membership degrees in the neutrosophic set lack the reliability measures related to them. If the Z-number notion is extended to the neutrosophic set, we can describe the hybrid information of combining the truth, falsity and indeterminacy degrees with their corresponding reliability degrees by three order pairs of fuzzy numbers. In multicriteria decision making (MDM) problems, the information expressions and decision making methods are vital research topics [39–42]. Motivated based on the ideas of combining the Z-number with the neutrosophic set and enhancing MDM reliability, the objects of this study are to present a more generalized neutrosophic notion closely related to reliability and to use it for MDM problems. To do so, this paper proposes the concept of a neutrosophic Z-number (NZN) set, which is a new framework of neutrosophic values combined with the neutrosophic measures of reliability, as the generalization of the Z-number and the neutrosophic set. Then, we define the operations of neutrosophic Z-numbers (NZNs) and a score function for ranking NZNs and propose NZN weighted arithmetic averaging (NZNWAA) and NZN weighted geometric averaging (NZNWGA) operators to aggregate NZN information. Regarding the NZNWAA and NZNWGA operators and the score function, a MDM approach is developed in the NZN environment. An illustrative example is used to demonstrate the applicability and effectiveness of the developed MDM approach in NZN setting. However, the proposed NZN notion and the developed MDM approach based on the NZNWAA and NZNWGA operators and the score function of NTN shows the novelty of this study.

For the first time study, the main contributions of the article are included as follows:

- The proposed NZN set can solve the information expression problem of the truth, falsity and indeterminacy values combined with their related reliability measures by the three order pairs of fuzzy numbers in indeterminate and inconsistent situations.
- The defined operations and NZNWAA and NZNWGA operators of NZNs are to realize the aggregation problems of the NZN information and then the score function of NZN is to rank NZNs, which provide the useful mathematical tools for MDM problems in NZN setting.
- The developed MDM approach not only enhances the MDM reliability but also provides a new effective way for MDM problems in NZN setting.

The study is organized as the following structures. Section “Neutrosophic Z-number set” presents the notion of a NZN set, operations of NZNs, and a score function of NZN for comparing NZNs. Section “Two weighted aggregation operators of neutrosophic Z-numbers” proposes the NZNWAA and NZNWGA operators and presents their properties. A MDM approach based on the NZNWAA and NZNWGA operators and the score function is developed in section “MDM approach using the NZNWAA and NZNWGA operators and the score function”. In section “An illustrative example and relative comparative analysis”, an illustrative example and the relative comparative analysis are presented to demonstrate the applicability and effectiveness of the developed MDM approach in NZN setting. Lastly, conclusions and further study are presented in section “Conclusion”.

### Neutrosophic Z-number set

In 2011, Zadeh [8] firstly introduced the concept of Z-number by an order pair of fuzzy numbers Z = (*V*, *R*) associated with a real-valued uncertain variable *X*, where the first component *V* is a fuzzy restriction on the values that *X* can take and the second component *R* is a measure of reliability for *V*.

Based on an extension of the Z-number concept [8] and the neutrosophic set, we can give the definition of a NZN set.

#### **Definition 1**

Set *X* as a universe set. Then a NZN set in a universe set *X* is defined as the following form:

$$ S_{Z} = \left\{ {\left\langle {x,T(V,R)(x),I(V,R)(x),F(V,R)(x)} \right\rangle |x \in X} \right\}, $$

where *T*(*V*, *R*)(*x*) = (*T*_*V*_(*x*), *F*_*R*_(*x*)), *I*(*V*, *R*)(*x*) = (*I*_*V*_(*x*), *I*_*R*_(*x*)), *F*(*V*, *R*)(*x*) = (*F*_*V*_(*x*), *F*_*R*_(*x*)): *X* → [0, 1]^2^ are the order pairs of truth, indeterminacy and falsity fuzzy values, then the first component *V* is neutrosophic values in a universe set *X* and the second component *R* is neutrosophic measures of reliability for *V*, along with the conditions $0 \le T_{V} (x) + I_{V} (x) + F_{V} (x) \le 3$ and $0 \le T_{R} (x) + I_{R} (x) + F_{R} (x) \le 3$.

For the convenient representation, the element $\left\langle {x,T(V,R)(x),I(V,R)(x),F(V,R)(x)} \right\rangle$ in *S*_*Z*_ is simply denoted as $s_{Z} = \left\langle {T(V,R),I(V,R),F(V,R)} \right\rangle = \left\langle {(T_{V} ,T_{R} ),(I_{V} ,I_{R} ),(F_{V} ,F_{R} )} \right\rangle$, which is named NZN.

#### **Definition 2**

Let $s_{Z1} = \left\langle {T_{1} (V,R),I_{1} (V,R),F_{1} (V,R)} \right\rangle = \left\langle {\left( {T_{V1} ,T_{R1} } \right),\left( {I_{V1} ,I_{R1} } \right),\left( {F_{V1} ,F_{R1} } \right)} \right\rangle$ and $s_{Z2} = \left\langle {T_{2} (V,R),I_{2} (V,R),F_{2} (V,R)} \right\rangle = \left\langle {\left( {T_{V2} ,T_{R2} } \right),\left( {I_{V2} ,I_{R2} } \right),\left( {F_{V2} ,F_{R2} } \right)} \right\rangle$ be two NZNs and λ > 0. Then, we give the following relations:

1. *s*_*Z*1_ ⊇ *s*_*Z*2_ ⇔ *T*_*V*1_ ≥ *T*_*V*2_, *T*_*R*1_ ≥ *T*_*R*2_, *I*_*V*1_ ≤ *I*_*V*2_, *I*_*R*1_ ≤ *I*_*R*2_, *F*_*V*1_ ≤ *F*_*V*2_, and *F*_*R*1_ ≤ *F*_*R*2_;
2. *s*_*Z*1_ = *s*_*Z*2_ ⇔ *s*_*Z*1_ ⊇ *s*_*Z*2_ and *s*_*Z*2_ ⊇ *s*_*Z*1_;
3. $$ s_{Z1} \cup s_{Z2} = \Big\langle (T_{V1}^{{}} \vee T_{V2}^{{}} ,T_{R1}^{{}} \vee T_{R2}^{{}} ),(I_{V1}^{{}} \wedge I_{V2}^{{}} ,I_{R1}^{{}} \wedge I_{R2}^{{}} ),(F_{V1}^{{}} \wedge F_{V2}^{{}} ,F_{R1}^{{}} \wedge F_{R2}^{{}} ) \Big\rangle $$;
4. $$ s_{Z1} \cap s_{Z2} = \Big\langle (T_{V1}^{{}} \wedge T_{V2}^{{}} ,T_{R1}^{{}} \wedge T_{R2}^{{}} ),(I_{V1}^{{}} \vee I_{V2}^{{}} ,I_{R1}^{{}} \vee I_{R2}^{{}} ),(F_{V1}^{{}} \vee F_{V2}^{{}} ,F_{R1}^{{}} \vee F_{R2}^{{}} ) \Big\rangle $$;
5. $$ (s_{Z1} )^{C} = \left\langle {\left( {F_{V1} ,F_{R1} } \right),\left( {1 - I_{V1} ,1 - I_{R1} } \right),\left( {T_{V1} ,T_{R1} } \right)} \right\rangle $$ (Complement of *s*_*Z*1_);
6. $$ s_{Z1} \oplus s_{Z2} = \Big\langle \left( {T_{V1} + T_{V2} - T_{V1} T_{V2} ,T_{R1} + T_{R2} - T_{R1} T_{R2} } \right),\left( {I_{V1} I_{V2} ,I_{R1} I_{R2} } \right),\left( {F_{V1} F_{V2} ,F_{R1} F_{R2} } \right) \Big\rangle $$;
7. $$ {s_{Z1}}  \otimes {s_{Z2}}  = \left\langle \begin{gathered}   \left( {{T_{V1}} {T_{V2}} ,{T_{R1}} {T_{R2}} } \right),\left( {{I_{V1}}  + {I_{V2}}  -{ I_{V1}} {I_{V2}}, {I_{R1}}  + {I_{R2}}  -{ I_{R1}} {I_{R2}} } \right), \hfill \\   \left( {{F_{V1}}  + {F_{V2}}  -{ F_{V1}} {F_{V2}} ,{F_{R1}}  + {F_{R2}}  - {F_{R1}} {F_{R2}} } \right) \hfill \\  \end{gathered}  \right\rangle  $$;
8. $$ \lambda s_{Z1} = \Big\langle \left( {1 - (1 - T_{V1} )^{\lambda } ,1 - (1 - T_{R1} )^{\lambda } } \right),\left( {I_{V1}^{\lambda } ,I_{R1}^{\lambda } } \right),\left( {F_{V1}^{\lambda } ,F_{R1}^{\lambda } } \right) \Big\rangle $$;
9. $$ s_{Z1}^{\lambda } = \Big\langle \left( {T_{V1}^{\lambda } ,T_{R1}^{\lambda } } \right),\left( {1 - (1 - I_{V1} )^{\lambda } ,1 - (1 - I_{R1} )^{\lambda } } \right),\left( {1 - (1 - F_{V1} )^{\lambda } ,1 - (1 - F_{R1} )^{\lambda } } \right) \Big\rangle $$.

To compare NZNs $s_{Zi} = \big\langle T_{i} (V,R),I_{i} (V,R),F_{i} (V,R) \big\rangle = \left\langle {\left( {T_{Vi} ,T_{Ri} } \right),\left( {I_{Vi} ,I_{Ri} } \right),\left( {F_{Vi} ,F_{Ri} } \right)} \right\rangle$ (*i* = 1, 2), we introduce a score function:

1 $$ Y(s_{Zi} ) = \frac{{2 + T_{Vi} T_{Ri} - I_{Vi} I_{Ri} - F_{Vi} F_{Ri} }}{3}\;\;{\text{for}}\;\;Y(s_{Zi} ) \in [0,1] $$

Thus, if *Y*(*s*_Z1_) ≥ *Y*(*s*_Z2_), there is the ranking *s*_Z1_ ≥ *s*_Z2_.

#### *Example 1*

Set two NZNs as *s*_*Z*1_ = < (0.7, 0.8), (0.1, 0.7), (0.3, 0.8) > and *s*_*Z*2_ = < (0.6, 0.9), (0.3, 0.8), (0.2, 0.7) > . Then, their ranking is given as follows:

By Eq. (1), we have *Y*(*s*_*Z*1_) = (2 + 0.7 × 0.8−0.1 × 0.7−0.3 × 0.8)/3 = 0.75 and *Y*(*s*_*Z*2_) = (2 + 0.6 × 0.9−0.3 × 0.8−0.2 × 0.7)/3 = 0.72. Since *Y*(*s*_*Z*1_) > *Y*(*s*_*Z*2_), their ranking is *s*_*Z*1_ > *s*_*Z*2_.

## Two weighted aggregation operators of neutrosophic Z-numbers

Based on the operations (6)–(9) in Definition 2, we can propose the two weighted aggregation operators of NZNs in this section.

### NZNWAA operator

Based on the operations (6) and (8) in Definition 2, we can present the NZNWAA operator of NZNs.

#### **Definition 3**

Let $s_{Zi} = \left\langle {T_{i} (V,R),I_{i} (V,R),F_{i} (V,R)} \right\rangle = \left\langle {\left( {T_{Vi} ,T_{Ri} } \right),\left( {I_{Vi} ,I_{Ri} } \right),\left( {F_{Vi} ,F_{Ri} } \right)} \right\rangle$ (*i *= 1, 2, …, *n*) be a group of NZNs and NZNWAA: *Ω*^*n*^ → *Ω*. Then, the NZNWAA operator is defined as

2 $${\rm NZNWAA}(s_{Z1} ,s_{Z2} , \cdots ,s_{Zn} ) = \sum\limits_{i = 1}^{n} {\lambda_{i} s_{Zi} } , $$

where *λ*_*i*_ (*i* = 1, 2, …, *n*) is the weight of *s*_*Zi*_ with 0 ≤ *λ*_*i*_ ≤ 1 and $\sum\nolimits_{i = 1}^{n} {\lambda_{i} = 1}$.

#### **Theorem 1**

Let $s_{Zi} = \left\langle {T_{i} (V,R),I_{i} (V,R),F_{i} (V,R)} \right\rangle = \left\langle {\left( {T_{Vi} ,T_{Ri} } \right),\left( {I_{Vi} ,I_{Ri} } \right),\left( {F_{Vi} ,F_{Ri} } \right)} \right\rangle$ (*i* = 1, 2, …, *n*) be a group of NZNs. Then, the collected value of the NZNWAA operator is a NZN, which is obtained by the following formula:

3 $$ \begin{aligned} & {\rm NZNWAA}(s_{Z1} ,s_{Z2} , \ldots ,s_{Zn} ) = \sum\limits_{i = 1}^{n} {\lambda_{i} s_{Zi} } \hfill \\ & \quad = \left\langle \left( {1 - \prod\limits_{i = 1}^{n} {(1 - T_{Vi}^{{}} )^{{\lambda_{i} }} } ,1 - \prod\limits_{i = 1}^{n} {(1 - T_{Ri}^{{}} )^{{\lambda_{i} }} } } \right), \right. \\ & \qquad \left. \left( {\prod\limits_{i = 1}^{n} {I_{Vi}^{{\lambda_{i} }} } ,\prod\limits_{i = 1}^{n} {I_{Ri}^{{\lambda_{i} }} } } \right),\left( {\prod\limits_{i = 1}^{n} {F_{Vi}^{{\lambda_{i} }} } ,\prod\limits_{i = 1}^{n} {F_{Ri}^{{\lambda_{i} }} } } \right) \right\rangle , \hfill \\ \end{aligned} $$

where *λ*_*i*_ is the weight of *s*_*Zi*_ (*i* = 1, 2, …, *n*) with 0 ≤ *λ*_*i*_ ≤ 1 and $\sum\nolimits_{i = 1}^{n} {\lambda_{i} = 1}$.

#### *Proof*

Regarding mathematical induction, Eq. (3) is verified below.

1. If *n* = 2, according to the operations (6) and (8) in Definition 2 we yield the following result:

4 $$ \begin{aligned} {\rm NZNWAA}(s_{Z1} ,s_{Z2} ) & = \lambda_{1} s_{Z1} \oplus \lambda_{2} s_{Z2} \\ & = \left\langle \begin{aligned} \left( {1 - (1 - T_{V1}^{{}} )^{{\lambda_{1} }} + 1 - (1 - T_{V2}^{{}} )^{{\lambda_{2} }} - \left( {1 - (1 - T_{V1}^{{}} )^{{\lambda_{1} }} } \right)\left( {1 - (1 - T_{V2}^{{}} )^{{\lambda_{2} }} } \right),} \right. \hfill \\ \left. {1 - (1 - T_{R1}^{{}} )^{{\lambda_{1} }} + 1 - (1 - T_{R2}^{{}} )^{{\lambda_{2} }} - \left( {1 - (1 - T_{R1}^{{}} )^{{\lambda_{1} }} } \right)\left( {1 - (1 - T_{R2}^{{}} )^{{\lambda_{2} }} } \right)} \right), \hfill \\ \left( {I_{V1}^{{\lambda_{1} }} I_{V2}^{{\lambda_{2} }} ,I_{R1}^{{\lambda_{1} }} I_{R2}^{{\lambda_{2} }} } \right),\left( {F_{V1}^{{\lambda_{1} }} F_{V2}^{{\lambda_{2} }} ,F_{R1}^{{\lambda_{1} }} F_{R2}^{{\lambda_{2} }} } \right) \hfill \\ \end{aligned} \right\rangle \\ & = \left\langle {\left( {1 - \prod\limits_{i = 1}^{2} {(1 - T_{Vi}^{{}} )^{{\lambda_{i} }} } ,1 - \prod\limits_{i = 1}^{2} {(1 - T_{Ri}^{{}} )^{{\lambda_{i} }} } } \right),\left( {\prod\limits_{i = 1}^{2} {I_{Vi}^{{\lambda_{i} }} } ,\prod\limits_{i = 1}^{2} {I_{Ri}^{{\lambda_{i} }} } } \right),\left( {\prod\limits_{i = 1}^{2} {F_{Vi}^{{\lambda_{i} }} } ,\prod\limits_{i = 1}^{2} {F_{Ri}^{{\lambda_{i} }} } } \right)} \right\rangle . \\ \end{aligned} $$

2. If *n* = *m*, Eq. (3) has the following form:

5 $$ \begin{aligned} &{\rm  NZNWAA}(s_{Z1} ,s_{Z2} , \ldots ,s_{Zm} ) = \sum\limits_{i = 1}^{m} {\lambda_{i} s_{Zi} } \\ & \quad = \left\langle \left( {1 - \prod\limits_{i = 1}^{m} {(1 - T_{Vi}^{{}} )^{{\lambda_{i} }} } ,1 - \prod\limits_{i = 1}^{m} {(1 - T_{Ri}^{{}} )^{{\lambda_{i} }} } } \right), \right. \\ &\qquad \left. \left( {\prod\limits_{i = 1}^{m} {I_{Vi}^{{\lambda_{i} }} } ,\prod\limits_{i = 1}^{m} {I_{Ri}^{{\lambda_{i} }} } } \right),\left( {\prod\limits_{i = 1}^{m} {F_{Vi}^{{\lambda_{i} }} } ,\prod\limits_{i = 1}^{m} {F_{Ri}^{{\lambda_{i} }} } } \right) \right\rangle . \\ \end{aligned} $$

3. If *n* = *m *+ 1, according to the operations (6) and (8) in Definition 2 and Eqs. (4) and (5), there is the following result:

$$ \begin{aligned} & {\rm NZNWAA}(s_{Z1} ,s_{Z2} , \ldots ,s_{Zm} ,s_{Zm + 1} )\\ &\quad = \sum\limits_{i = 1}^{m} {\lambda_{i} s_{Zi} } \oplus \lambda_{m + 1} s_{Zm + 1} \\ &\quad  = \left\langle \left( {1 - \prod\limits_{i = 1}^{m} {(1 - T_{Vi}^{{}} )^{{\lambda_{i} }} } ,1 - \prod\limits_{i = 1}^{m} {(1 - T_{Ri}^{{}} )^{{\lambda_{i} }} } } \right), \right. \\ &\qquad \left. \left( {\prod\limits_{i = 1}^{m} {I_{Vi}^{{\lambda_{i} }} } ,\prod\limits_{i = 1}^{m} {I_{Ri}^{{\lambda_{i} }} } } \right),\left( {\prod\limits_{i = 1}^{m} {F_{Vi}^{{\lambda_{i} }} } ,\prod\limits_{i = 1}^{m} {F_{Ri}^{{\lambda_{i} }} } } \right) \right\rangle \oplus \lambda_{m + 1} s_{Zm + 1} \\ &\quad  = \left\langle \left( {1 - \prod\limits_{i = 1}^{m + 1} {(1 - T_{Vi}^{{}} )^{{\lambda_{i} }} } ,1 - \prod\limits_{i = 1}^{m + 1} {(1 - T_{Ri}^{{}} )^{{\lambda_{i} }} } } \right), \right. \\ &\qquad \left. \left( {\prod\limits_{i = 1}^{m + 1} {I_{Vi}^{{\lambda_{i} }} } ,\prod\limits_{i = 1}^{m + 1} {I_{Ri}^{{\lambda_{i} }} } } \right),\left( {\prod\limits_{i = 1}^{m + 1} {F_{Vi}^{{\lambda_{i} }} } ,\prod\limits_{i = 1}^{m + 1} {F_{Ri}^{{\lambda_{i} }} } } \right) \right\rangle . \\ \end{aligned} $$

Based on the above results, Eq. (3) can keep for any *n*.

Thus, the verification is finished.□

#### **Theorem 2**

The NZNWAA operator of Eq. (3) implies the following properties:

1. Idempotency: Set $s_{Zi} = \left\langle {T_{i} (V,R),I_{i} (V,R),F_{i} (V,R)} \right\rangle = \left\langle {\left( {T_{Vi} ,T_{Ri} } \right),\left( {I_{Vi} ,I_{Ri} } \right),\left( {F_{Vi} ,F_{Ri} } \right)} \right\rangle$ (*i* = 1, 2,…, *n*) as a group of NZNs. If *s*_*Zi*_= *s*_*Z*_ (*i* = 1, 2,…, *n*), there is ${\rm NZNWAA}\left( {s_{Z1} ,s_{Z2} , \cdots ,s_{Zn} } \right) = s_{Z}$.
2. Boundedness: Set $s_{Zi} = \left\langle {T_{i} (V,R),I_{i} (V,R),F_{i} (V,R)} \right\rangle = \left\langle {\left( {T_{Vi} ,T_{Ri} } \right),\left( {I_{Vi} ,I_{Ri} } \right),\left( {F_{Vi} ,F_{Ri} } \right)} \right\rangle$ (*i* = 1, 2, …, *n*) as a group of NZNs and let

$$ \begin{aligned}  s_{Z\hbox{min} } &= \left\langle {\mathop {\hbox{min} }\limits_{i} \left( {T_{i} (V,R)} \right),\mathop {\rm max}\limits_{i} \left( {I_{i} (V,R)} \right),\mathop {\rm max}\limits_{i} \left( {F_{i} (V,R)} \right)} \right\rangle \hfill \\ &  = \left\langle \left( {\mathop {\hbox{min} }\limits_{i} \left( {T_{Vi}^{{}} } \right),\mathop {\hbox{min} }\limits_{i} \left( {T_{Ri}^{{}} } \right)} \right),\left( {\mathop {\hbox{max} }\limits_{i} \left( {I_{Vi}^{{}} } \right),\mathop {\hbox{max} }\limits_{i} \left( {I_{Ri}^{{}} } \right)} \right), \right. \\ &\qquad \left. \left( {\mathop {\hbox{max} }\limits_{i} \left( {F_{Vi}^{{}} } \right),\mathop {\hbox{max} }\limits_{i} \left( {F_{Ri}^{{}} } \right)} \right) \right\rangle , \hfill \\ \end{aligned} $$

$$ \begin{aligned} s_{Z\hbox{max} } &= \left\langle {\mathop {\hbox{max} }\limits_{i} \left( {T_{i} (V,R)} \right),\mathop {\hbox{min} }\limits_{i} \left( {I_{i} (V,R)} \right),\mathop {\hbox{min} }\limits_{i} \left( {F_{i} (V,R)} \right)} \right\rangle \hfill \\ &   = \left\langle \left( {\mathop {\hbox{max} }\limits_{i} \left( {T_{Vi}^{{}} } \right),\mathop {\hbox{max} }\limits_{i} \left( {T_{Ri}^{{}} } \right)} \right),\left( {\mathop {\hbox{min} }\limits_{i} \left( {I_{Vi}^{{}} } \right),\mathop {\hbox{min} }\limits_{i} \left( {I_{Ri}^{{}} } \right)} \right), \right. \\ &\qquad \left. \left( {\mathop {\hbox{min} }\limits_{i} \left( {F_{Vi}^{{}} } \right),\mathop {\hbox{min} }\limits_{i} \left( {F_{Ri}^{{}} } \right)} \right) \right\rangle . \hfill \\ \end{aligned} $$

Then, $s_{Z\hbox{min} } \le {\rm NZNWAA}(s_{Z1} ,s_{Z2} , \cdots ,s_{Zn} ) \le s_{Z\hbox{max} }$ can keep.

3. Monotonicity: Set $s_{Zi} = \big\langle T_{i} (V,R),I_{i} (V,R),F_{i} (V,R) \big\rangle = \left\langle {\left( {T_{Vi} ,T_{Ri} } \right),\left( {I_{Vi} ,I_{Ri} } \right),\left( {F_{Vi} ,F_{Ri} } \right)} \right\rangle$ and $s_{Zi}^{*} = \left\langle {T_{i}^{*} (V,R),I_{i}^{*} (V,R),F_{i}^{*} (V,R)} \right\rangle = \left\langle {\left( {T_{Vi}^{*} ,T_{Ri}^{*} } \right),\left( {I_{Vi}^{*} ,I_{Ri}^{*} } \right),\left( {F_{Vi}^{*} ,F_{Ri}^{*} } \right)} \right\rangle$ (*i* = 1, 2, …, *n*) as two groups of NZNs. When $s_{Zi} \le s_{Zi}^{*}$, there is ${\rm  NZNWAA}(s_{Z1} ,s_{Z2} , \cdots ,s_{Zn} ) \le NZNWAA(s_{Z1}^{*} ,s_{Z2}^{*} , \cdots ,s_{Zn}^{*} )$.

#### *Proof*

1. If *s*_*Zi*_ = *s*_*Z*_ (*i* = 1, 2,…, *n*), the result of Eq. (3) is given by

$$ \begin{aligned}  &{\rm NZNWAA  }\left( {s_{Z1} ,s_{Z2} , \ldots ,s_{Zn} } \right) = \sum\limits_{i = 1}^{n} {\lambda_{i} s_{Zi} } \\ &\quad = \left\langle \left( {1 - \prod\limits_{i = 1}^{n} {(1 - T_{Vi}^{{}} )^{{\lambda_{i} }} } ,1 - \prod\limits_{i = 1}^{n} {(1 - T_{Ri}^{{}} )^{{\lambda_{i} }} } } \right),   \right. \\ &\qquad \left.  \left( {\prod\limits_{i = 1}^{n} {I_{Vi}^{{\lambda_{i} }} } ,\prod\limits_{i = 1}^{n} {I_{Ri}^{{\lambda_{i} }} } } \right),\left( {\prod\limits_{i = 1}^{n} {F_{Vi}^{{\lambda_{i} }} } ,\prod\limits_{i = 1}^{n} {F_{Ri}^{{\lambda_{i} }} } } \right) \right\rangle \\ &\quad = \left\langle \left( {1 - (1 - T_{V}^{{}} )^{{\sum\nolimits_{i = 1}^{n} {\lambda_{i} } }} ,1 - (1 - T_{R}^{{}} )^{{\sum\nolimits_{i = 1}^{n} {\lambda_{i} } }} } \right), \right. \\ &\qquad \left. \left( {(I_{V} )_{{}}^{{\sum\nolimits_{i = 1}^{n} {\lambda_{i} } }} ,(I_{R} )_{{}}^{{\sum\nolimits_{i = 1}^{n} {\lambda_{i} } }} } \right),\left( {(F_{V} )_{{}}^{{\sum\nolimits_{i = 1}^{n} {\lambda_{i} } }} ,(F_{R} )_{{}}^{{\sum\nolimits_{i = 1}^{n} {\lambda_{i} } }} } \right) \right\rangle \\ &\quad  = \left\langle {\left( {1 - (1 - T_{V}^{{}} ),1 - (1 - T_{R}^{{}} )} \right),\left( {I_{V} ,I_{R} } \right),\left( {F_{V} ,F_{R} } \right)} \right\rangle \\ &\quad = \left\langle {\left( {T_{V} ,T_{R} } \right),\left( {I_{V} ,I_{R} } \right),\left( {F_{V} ,F_{R} } \right)} \right\rangle = s_{Z} . \\ \end{aligned} $$

2. Since *s*_*Zmin*_ and *s*_*Zmax*_ are given by the minimum NZN and the maximum NZN, the inequality *s*_*Zmin*_ ≤ *s*_*Zi*_ ≤ *s*_*Zmax*_ exists. Thus, there is $\sum\nolimits_{i = 1}^{n} {\lambda_{i} s_{Z\hbox{min} } } \le \sum\nolimits_{i = 1}^{n} {\lambda_{i} s_{Zi} } \le \sum\nolimits_{i = 1}^{n} {\lambda_{i} s_{Z\hbox{max} } }$. Based on the above property (1), $s_{Z\hbox{min} } \le \sum\nolimits_{i = 1}^{n} {\lambda_{i} s_{Zi} \le } s_{Z\hbox{max} }$ can exist, i.e., there is $s_{Z\hbox{min} } \le NZNWAA\left( {s_{Z1} ,s_{Z2} , \cdots ,s_{Zn} } \right) \le s_{Z\hbox{max} }$.
3. Since $s_{Zi} \le s_{Zi}^{*}$, there is $\sum\nolimits_{i = 1}^{n} {\lambda_{i} s_{Zi} } \le \sum\nolimits_{i = 1}^{n} {\lambda_{i} s_{Zi}^{*} }$, i.e., ${\rm NZNWAA}\left( {s_{Z1} ,s_{Z2} , \cdots ,s_{Zn} } \right) \le NZNWAA\left( {s_{Z1}^{*} ,s_{Z2}^{*} , \cdots ,s_{Zn}^{*} } \right)$.

Thus, the verification of all properties is completed.□

### NZNWGA operator

Based on the operations (7) and (9) in Definition 2, we can present the NZNWGA operator of NZNs.

#### **Definition 4**

Let $s_{Zi} = \left\langle {T_{i} (V,R),I_{i} (V,R),F_{i} (V,R)} \right\rangle = \left\langle {\left( {T_{Vi} ,T_{Ri} } \right),\left( {I_{Vi} ,I_{Ri} } \right),\left( {F_{Vi} ,F_{Ri} } \right)} \right\rangle$ (*i *= 1, 2, …, *n*) be a group of NZNs and NZNWGA: *Ω*^*n*^ → *Ω*. Then, the NZNWGA operator is defined as

6 $$ {\rm NZNWGA}(s_{Z1} ,s_{Z2} , \ldots ,s_{Zn} ) = \prod\limits_{i = 1}^{n} {s_{Zi}^{{\lambda_{i} }} } , $$

where *λ*_*i*_ (*i* = 1, 2, …, *n*) is the weight of *s*_*Zi*_ with 0 ≤ *λ*_*i*_ ≤ 1 and $\sum\nolimits_{i = 1}^{n} {\lambda_{i} = 1}$.

#### **Theorem 3**

Let $s_{Zi} = \left\langle {T_{i} (V,R),I_{i} (V,R),F_{i} (V,R)} \right\rangle = \left\langle {\left( {T_{Vi} ,T_{Ri} } \right),\left( {I_{Vi} ,I_{Ri} } \right),\left( {F_{Vi} ,F_{Ri} } \right)} \right\rangle$ (*i* = 1, 2, …, *n*) be a group of NZNs. Then, the collected value of the NZNWGA operator is a NZN, which is obtained by the following formula:

7 $$ \begin{aligned} &{\rm NZNWGA}(s_{Z1} ,s_{Z2} , \ldots ,s_{Zn} ) = \prod\limits_{i = 1}^{n} {s_{Zi}^{{\lambda_{i} }} } \\ &\quad = \left\langle \begin{aligned} \left( {\prod\limits_{i = 1}^{n} {T_{Vi}^{{\lambda_{i} }} } ,\prod\limits_{i = 1}^{n} {T_{Ri}^{{\lambda_{i} }} } } \right),\left( {1 - \prod\limits_{i = 1}^{n} {(1 - I_{Vi}^{{}} )^{{\lambda_{i} }} } ,1 - \prod\limits_{i = 1}^{n} {(1 - I_{Ri}^{{}} )^{{\lambda_{i} }} } } \right), \hfill \\ \left( {1 - \prod\limits_{i = 1}^{n} {(1 - F_{Vi}^{{}} )^{{\lambda_{i} }} } ,1 - \prod\limits_{i = 1}^{n} {(1 - F_{Ri}^{{}} )^{{\lambda_{i} }} } } \right) \hfill \\ \end{aligned} \right\rangle ,\end{aligned}  $$

where *λ*_*i*_ is the weight of *s*_*Zi*_ (*i* = 1, 2, …, *n*) with 0 ≤ *λ*_*i*_ ≤ 1 and $\sum\nolimits_{i = 1}^{n} {\lambda_{i} = 1}$.

By the similar verification process of Theorem 1, we can also verify that the NZNWGA operator of Eq. (7) is true, which is not repeated here.

#### **Theorem 4**

The NZNWGA operator of Eq. (7) also implies the following properties:

1. Idempotency: Set $s_{Zi} = \left\langle {T_{i} (V,R),I_{i} (V,R),F_{i} (V,R)} \right\rangle = \left\langle {\left( {T_{Vi} ,T_{Ri} } \right),\left( {I_{Vi} ,I_{Ri} } \right),\left( {F_{Vi} ,F_{Ri} } \right)} \right\rangle$ (*i* = 1, 2,…, *n*) as a group of NZNs. If *s*_*Zi*_= *s*_*Z*_ (*i* = 1, 2,…, *n*), there is ${\rm NZNWGA}\left( {s_{Z1} ,s_{Z2} , \cdots ,s_{Zn} } \right) = s_{Z}$.
2. Boundedness: Set $s_{Zi} = \big\langle T_{i} (V,R),I_{i} (V,R),F_{i} (V,R) \big\rangle = \left\langle {\left( {T_{Vi} ,T_{Ri} } \right),\left( {I_{Vi} ,I_{Ri} } \right),\left( {F_{Vi} ,F_{Ri} } \right)} \right\rangle$ (*i* = 1, 2, …, *n*) as a group of NZNs and let

$$ \begin{aligned} s_{Z\hbox{min} } & = \left\langle {\mathop {\hbox{min} }\limits_{i} \left( {T_{i} (V,R)} \right),\mathop {\rm max}\limits_{i} \left( {I_{i} (V,R)} \right),\mathop {\rm max}\limits_{i} \left( {F_{i} (V,R)} \right)} \right\rangle \\ & = \left\langle \left( {\mathop {\hbox{min} }\limits_{i} \left( {T_{Vi}^{{}} } \right),\mathop {\hbox{min} }\limits_{i} \left( {T_{Ri}^{{}} } \right)} \right),\left( {\mathop {\hbox{max} }\limits_{i} \left( {I_{Vi}^{{}} } \right),\mathop {\hbox{max} }\limits_{i} \left( {I_{Ri}^{{}} } \right)} \right), \right. \\ &\quad \left. \left( {\mathop {\hbox{max} }\limits_{i} \left( {F_{Vi}^{{}} } \right),\mathop {\hbox{max} }\limits_{i} \left( {F_{Ri}^{{}} } \right)} \right) \right\rangle \\ \end{aligned} $$

$$ \begin{aligned} s_{Z\hbox{max} } & = \left\langle {\mathop {\hbox{max} }\limits_{i} \left( {T_{i} (V,R)} \right),\mathop {\hbox{min} }\limits_{i} \left( {I_{i} (V,R)} \right),\mathop {\hbox{min} }\limits_{i} \left( {F_{i} (V,R)} \right)} \right\rangle \\ &\quad  = \left\langle \left( {\mathop {\hbox{max} }\limits_{i} \left( {T_{Vi}^{{}} } \right),\mathop {\hbox{max} }\limits_{i} \left( {T_{Ri}^{{}} } \right)} \right),\left( {\mathop {\hbox{min} }\limits_{i} \left( {I_{Vi}^{{}} } \right),\mathop {\hbox{min} }\limits_{i} \left( {I_{Ri}^{{}} } \right)} \right), \right. \\ &\qquad \left. \left( {\mathop {\hbox{min} }\limits_{i} \left( {F_{Vi}^{{}} } \right),\mathop {\hbox{min} }\limits_{i} \left( {F_{Ri}^{{}} } \right)} \right) \right\rangle . \\ \end{aligned} $$

Then, $s_{Z\hbox{min} } \le NZNWGA(s_{Z1} ,s_{Z2} , \ldots ,s_{Zn} ) \le s_{Z\hbox{max} }$ can keep.

3. Monotonicity: Set $s_{Zi} = \big\langle T_{i} (V,R),I_{i} (V,R),F_{i} (V,R) \big\rangle = \left\langle {\left( {T_{Vi} ,T_{Ri} } \right),\left( {I_{Vi} ,I_{Ri} } \right),\left( {F_{Vi} ,F_{Ri} } \right)} \right\rangle$ and $s_{Zi}^{*} = \left\langle {T_{i}^{*} (V,R),I_{i}^{*} (V,R),F_{i}^{*} (V,R)} \right\rangle = \left\langle {\left( {T_{Vi}^{*} ,T_{Ri}^{*} } \right),\left( {I_{Vi}^{*} ,I_{Ri}^{*} } \right),\left( {F_{Vi}^{*} ,F_{Ri}^{*} } \right)} \right\rangle$ (*i* = 1, 2, …, *n*) as two groups of NZNs. When $s_{Zi} \le s_{Zi}^{*}$, there is ${\rm NZNWGA}(s_{Z1} ,s_{Z2} , \ldots ,s_{Zn} ) \le NZNWGA\left( {s_{Z1}^{*} ,s_{Z2}^{*} , \ldots ,s_{Zn}^{*} } \right)$.

Obviously, the above properties corresponding to the NZNWGA operator can be also verified by the similar verification process of Theorem 2, which is not repeated here.

## MDM approach using the NZNWAA and NZNWGA operators and the score function

Regarding the proposed NZNWAA and NZNWGA operators and the score function, this section develops a MDM approach to solve MDM problems with the evaluation information of both neutrosophic values and neutrosophic measures of reliability related to the neutrosophic values in NZN setting.

Suppose that in a MDM problem a set of *m* alternatives *Q* = {*Q*_1_, *Q*_2_, …, *Q*_*m*_} is presented and assessed by a set of *n* criteria *X* = {*x*_1_, *x*_2_, …, *x*_*n*_}. Then, the impotence of each criterion *x*_*i*_ (*i *= 1, 2, …, *n*) is considered by the weight *λ*_*i*_, which is constructed as the weight vector ***λ ***= (*λ*_1_, *λ*_2_, …, *λ*_*n*_). Decision makers are requested to give the suitability assessment of each criterion *x*_*i*_ (*i* = 1, 2, …, *n*) for each alternative *Q*_*j*_ (*j* = 1, 2, …, *m*) by both the truth, falsity, indeterminacy fuzzy values and the measures of corresponding reliabilities, which are constructed as a NZN $s_{Zji} = \left\langle {T_{ji} (V,R),I_{ji} (V,R),F_{ji} (V,R)} \right\rangle = \left\langle {\left( {T_{Vji} ,T_{Rji} } \right),\left( {I_{Vji} ,I_{Rji} } \right),\left( {F_{Vji} ,F_{Rji} } \right)} \right\rangle$, where $T_{Vji} ,I_{Vji} ,F_{Vji} \in [0,1]$ and $T_{Rji} ,I_{Rji} ,F_{Rji} \in [0,1]$. Thus, the decision matrix of NZNs can be represented as *S*_*Z*_ = (*s*_*Zji*_)_*m*×*n*_. In this MDM problem, the decision process is described below:

Step 1: Based on Eq. (3) or Eq. (7), the overall NZN *s*_*Zj*_ is obtained by

8 $$ \begin{aligned} s_{Zj} & ={\rm NZNWAA}(s_{Zj1} ,s_{Zj2} , \ldots ,s_{Zjn} ) = \sum\limits_{i = 1}^{n} {\lambda_{i} s_{Zji} } \\ &\quad  = \left\langle \left( {1 - \prod\limits_{i = 1}^{n} {(1 - T_{Vji}^{{}} )^{{\lambda_{i} }} } ,1 - \prod\limits_{i = 1}^{n} {(1 - T_{Rji}^{{}} )^{{\lambda_{i} }} } } \right), \right. \\ &\qquad \left. \left( {\prod\limits_{i = 1}^{n} {I_{Vji}^{{\lambda_{i} }} } ,\prod\limits_{i = 1}^{n} {I_{Rji}^{{\lambda_{i} }} } } \right),\left( {\prod\limits_{i = 1}^{n} {F_{Vji}^{{\lambda_{i} }} } ,\prod\limits_{i = 1}^{n} {F_{Rji}^{{\lambda_{i} }} } } \right) \right\rangle \\ \end{aligned} $$

or

9 $$ \begin{aligned} s_{Zj} & = {\rm NZNWGA}(s_{Zj1} ,s_{Zj2} , \ldots ,s_{Zjn} ) = \prod\limits_{i = 1}^{n} {s_{Zji}^{{\lambda_{i} }} } \\ & = \left\langle \left( {\prod\limits_{i = 1}^{n} {T_{Vji}^{{\lambda_{i} }} } ,\prod\limits_{i = 1}^{n} {T_{Rji}^{{\lambda_{i} }} } } \right), \right. \\ &\quad \left. \left( {1 - \prod\limits_{i = 1}^{n} {(1 - I_{Vji}^{{}} )^{{\lambda_{i} }} } ,1 - \prod\limits_{i = 1}^{n} {(1 - I_{Rji}^{{}} )^{{\lambda_{i} }} } } \right), \right. \\ &\quad \left.  \left( {1 - \prod\limits_{i = 1}^{n} {(1 - F_{Vji}^{{}} )^{{\lambda_{i} }} } ,1 - \prod\limits_{i = 1}^{n} {(1 - F_{Rji}^{{}} )^{{\lambda_{i} }} } } \right) \right\rangle \\ \end{aligned} .$$

Step 2: The score values of *Y*(*s*_*Zj*_) (*j* = 1, 2, …, *m*) are calculated using Eq. (1)
Step 3: The alternatives are ranked based on the score values and the best one is chosen among ones
Step 4: End

## An illustrative example and relative comparative analysis

### An illustrative example

This part provides an illustrative example about the selection problem of business partners to demonstrate the applicability and effectiveness of the developed MDM approach with NZN information.

Suppose a manufacturing company needs to choose a suitable supplier in potential business partners. The expert panel provides a set of four suppliers/alternatives *Q* = {*Q*_1_, *Q*_2_, *Q*_3_, *Q*_4_} from potential business partners, which must satisfy the assessment requirements of the three criteria: (1) *x*_1_ is the cost of product; (2) *x*_2_ is the quality of product; (3) *x*_3_ is the quality of service. The weigh vector of the three criteria is specified as ***λ***= (0.33, 0.35, 0.32) to indicate the importance of the three criteria. Then, the experts/decision makers are invited to assess the four suppliers/alternatives over the three criteria by the NZNs that are composed of their truth, falsity, indeterminacy fuzzy values and the measures of corresponding reliabilities. Thus, all NZNs can be constructed as the following NZN decision matrix:

$$ S_{Z} = (s_{Zji} )_{4 \times 3} = \left[ {\begin{array}{*{20}c} {\left\langle {(0.6,0.8),(0.1,0.7),(0.2,0.8)} \right\rangle } & {\left\langle {(0.7,0.6),(0.1,0.8),(0.2,0.7)} \right\rangle } & {\left\langle {(0.7,0.7),(0.2,0.8),(0.2,0.9)} \right\rangle } \\ {\left\langle {(0.8,0.7),(0.1,0.8),(0.2,0.6)} \right\rangle } & {\left\langle {(0.6,0.7),(0.1,0.7),(0.2,0.7)} \right\rangle } & {\left\langle {(0.8,0.8),(0.4,0.7),(0.2,0.8)} \right\rangle } \\ {\left\langle {(0.6,0.6),(0.2,0.6),(0.1,0.7)} \right\rangle } & {\left\langle {(0.7,0.8),(0.2,0.7),(0.3,0.8)} \right\rangle } & {\left\langle {(0.7,0.7),(0.3,0.8),(0.6,0.7)} \right\rangle } \\ {\left\langle {(0.7,0.8),(0.1,0.7),(0.1,0.7)} \right\rangle } & {\left\langle {(0.6,0.7),(0.1,0.7),(0.1,0.9)} \right\rangle } & {\left\langle {(0.7,0.6),(0.2,0.7),(0.3,0.8)} \right\rangle } \\ \end{array} } \right]. $$

On the one hand, the developed MDM approach using the NZNWAA operator can be used for this MDM problem and depicted by the following decision process:

Step 1: Using Eq. (8), the overall collected NZNs *s*_*Zj*_ (*j* = 1, 2, 3, 4) are given as follows:

$$ \begin{aligned} s_{Z1} \, & = \, < \,\left( {0. 6 70 1, \, 0. 70 9 8} \right), \, \left( {0. 1 2 4 8, \, 0. 7 6 5 5} \right), \\ &\quad \left( {0. 2000, \, 0. 7 9 2 8} \right)\, > \,, \hfill \\ s_{Z2} & = \, < \,\left( {0. 7 4 5 1, \, 0. 7 3 6 5} \right), \, \left( {0. 1 5 5 8, \, 0. 7 3 1 5} \right), \\ &\quad  \left( {0. 2000, \, 0. 6 9 4 3} \right)\, > \,, \hfill \\ s_{Z3} & = \, < \,\left( {0. 6 70 1, \, 0. 7 1 3 8} \right), \, \left( {0. 2 2 7 7, \, 0. 6 9 4 3} \right), \\ &\quad  \left( {0. 2 60 6, \, 0. 7 3 3 5} \right)\, > \,, \hfill \\ s_{Z4} & = \, < \,\left( {0. 6 6 8 2, \, 0. 7 1 2 3} \right), \, \left( {0. 1 2 4 8, \, 0. 7000} \right), \\ &\quad  \left( {0. 1 4 2 1, \, 0. 7 9 7 7} \right)\, > \,. \hfill \\ \end{aligned} $$

Step 2: By Eq. (1), the score values of *Y*(*s*_*Zj*_) for the alternative *Q*_*j*_ (*j* = 1, 2, 3, 4) are yielded below:

$$ \begin{aligned} Y\left( {s_{Z1} } \right) & = \,0. 7 40 5,\;\;Y\left( {s_{Z2} } \right)\, = \,0. 7 6 5 3,\\ Y\left( {s_{Z3} } \right) & = \,0. 70 9 7, \, \;\;{\text{and}}\;Y\left( {s_{Z4} } \right)\, = \,0. 7 5 8 4. \end{aligned} $$

Step 3: According to the score values *Y*(*s*_*Z*2_) > *Y*(*s*_*Z*4_) > *Y*(*s*_*Z*1_) > *Y*(*s*_*Z*3_), the four alternatives are ranked as *Q*_2_ > *Q*_4_ > *Q*_1_ > *Q*_3_. Hence, the best supplier is *Q*_2_

On the other hand, the developed MDM approach using the NZNWGA operator can be also used for this MDM problem and depicted by the following decision process:

Step 1’: By Eq. (9), the overall collected NZNs *s*_*Zj*_ (*j* = 1, 2, 3, 4) are obtained as follows:

$$ \begin{aligned} s_{Z1} & = \, < \,\left( {0. 6 6 5 3, \, 0. 6 9 3 1} \right), \, \left( {0. 1 3 3 3, \, 0. 7 7 1 4} \right), \\ &\quad  \left( {0. 2000, \, 0. 8 1 5 4} \right)\, > \,, \hfill \\ s_{Z2} & = \, < \,\left( {0. 7 2 3 4, \, 0. 7 30 6} \right), \, \left( {0. 20 9 5, \, 0. 7 3 7 6} \right), \\ &\quad \left( {0. 2000, \, 0. 7 10 3} \right)\, > \,, \hfill \\ s_{Z3} &  = \, < \,\left( {0. 6 6 5, \, 0. 6 9 7 1} \right), \, \left( {0. 2 3 3 5, \, 0. 7 10 3} \right), \\ &\quad \left( {0. 3 6 4 2, \, 0. 7 3 9 7} \right)\, > \,, \hfill \\ s_{Z4} & = \, < \,\left( {0. 6 6 3 2, \, 0. 6 9 6 3} \right), \, \left( {0. 1 3 3 3, \, 0. 7000} \right), \\ &\quad \left( {0. 1 6 9 5, \, 0. 8 20 6} \right)\, > \,. \hfill \\ \end{aligned} $$

Step 2’: By Eq. (1), the score values of *Y*(*s*_*Zj*_) for the alternative *Q*_*j*_ (*j* = 1, 2, 3, 4) are given as follows:

$$ \begin{aligned} Y\left( {s_{Z1} } \right)\, & = \,0. 7 3 1 7,Y\left( {s_{Z2} } \right)\, = \,0. 7 4 40,\\ Y\left( {s_{Z3} } \right) & = \,0. 6 7 6 2, \, \;{\text{and}}\;\;Y\left( {s_{Z4} } \right)\, = \,0. 7 4 3 1. \end{aligned} $$

Step 3’: According to the score values *Y*(*s*_*Z*2_) > *Y*(*s*_*Z*4_) >  *Y*(*s*_*Z*1_) > *Y*(*s*_*Z*3_), the four alternatives are ranked as *Q*_2_ > *Q*_4_ > *Q*_1_ > *Q*_3_. Thus the best supplier is *Q*_2_

Based on the developed MDM approach using the NZNWAA and NZNWGA operators and the score function, we can see that the above two kinds of ranking orders regarding the four alternatives and the best one are identical. Hence, the developed MDM approach is effective.

### Relative comparison

For convenient comparison with existing relative method [33], assume we do not consider the assessment measures of corresponding reliabilities in the decision matrix *S*_*Z*_ as a special case of the above example. Then, the NZN decision matrix is reduced to the following single-valued neutrosophic decision matrix:

$$ S = (s_{ji} )_{4 \times 3} = \left[ {\begin{array}{*{20}c} {\left\langle {0.6,0.1,0.2} \right\rangle } & {\left\langle {0.7,0.1,0.2} \right\rangle } & {\left\langle {0.7,0.2,0.2} \right\rangle } \\ {\left\langle {0.8,0.1,0.2} \right\rangle } & {\left\langle {0.6,0.1,0.2} \right\rangle } & {\left\langle {0.8,0.4,0.2} \right\rangle } \\ {\left\langle {0.6,0.2,0.1} \right\rangle } & {\left\langle {0.7,0.2,0.3} \right\rangle } & {\left\langle {0.7,0.3,0.6} \right\rangle } \\ {\left\langle {0.7,0.1,0.1} \right\rangle } & {\left\langle {0.6,0.1,0.1} \right\rangle } & {\left\langle {0.7,0.2,0.3} \right\rangle } \\ \end{array} } \right] $$

Then, Eqs. (1), (8) and (9) are also reduced to existing score function, single-valued neutrosophic weighted arithmetic averaging (SVNWAA) and the single-valued neutrosophic weighted geometric averaging (SVNWGA) operators [33]:

10 $$ Y(s_{j} ) = \frac{{2 + T_{Vj} - I_{Vj} - F_{Vj} }}{3}\;\;{\text{for}}\;\;\;Y(s_{j} ) \in [0,1], $$

11 $$ \begin{aligned} s_{j} & = {\rm SVNWAA}(s_{j1} ,s_{j2} , \ldots ,s_{jn} )\; = \sum\limits_{i = 1}^{n} {\lambda_{i} s_{ji} } \\ & = \left\langle {1 - \prod\limits_{i = 1}^{n} {(1 - T_{Vji}^{{}} )^{{\lambda_{i} }} } ,\prod\limits_{i = 1}^{n} {I_{Vji}^{{\lambda_{i} }} } ,\prod\limits_{i = 1}^{n} {F_{Vji}^{{\lambda_{i} }} } } \right\rangle , \end{aligned} $$

12 $$ \begin{aligned} s_{j} & = {\rm SVNWGA}(s_{j1} ,s_{j2} , \ldots ,s_{jn} ) = \prod\limits_{i = 1}^{n} {s_{ji}^{{\lambda_{i} }} } \\ & = \left\langle {\prod\limits_{i = 1}^{n} {T_{Vji}^{{\lambda_{i} }} } ,1 - \prod\limits_{i = 1}^{n} {(1 - I_{Vji}^{{}} )^{{\lambda_{i} }} } ,1 - \prod\limits_{i = 1}^{n} {(1 - F_{Vji}^{{}} )^{{\lambda_{i} }} } } \right\rangle . \end{aligned} $$

Based on existing MDM method [33], decision results are given using Eqs. (10)–(12). Thus, all decision results obtained by existing MDM method [33] and the proposed MDM approach are shown in Table 1.

**Table 1:** Decision results based on various weighted aggregation operators

MDM method	Aggregated value	Score value	Ranking
MDM method based on the NZNWAA operator	*s*_*Z*1_ = < (0.6701, 0.7098), (0.1248, 0.7655), (0.2000, 0.7928) > , *s*_*Z*2_ = < (0.7451, 0.7365), (0.1558, 0.7315), (0.2000, 0.6943) > , *s*_*Z*3_ = < (0.6701, 0.7138), (0.2277, 0.6943), (0.2606, 0.7335) > , *s*_*Z*4_ = < (0.6682, 0.7123), (0.1248, 0.7000), (0.1421, 0.7977)>	*Y*(*s*_*Z*1_) = 0.7405, *Y*(*s*_*Z*2_) = 0.7653, *Y*(*s*_*Z*3_) = 0.7097, *Y*(*s*_*Z*4_) = 0.7584	*Q*_2_ > *Q*_4_ > *Q*_1_ > *Q*_3_
MDM method based on the NZNWGA operator	*s*_*Z*1_ = < (0.6653, 0.6931), (0.1333, 0.7714), (0.2000, 0.8154) > , *s*_*Z*2_ = < (0.7234, 0.7306), (0.2095, 0.7376), (0.2000, 0.7103) > , *s*_*Z*3_ = < (0.665, 0.6971), (0.2335, 0.7103), (0.3642, 0.7397) > , *s*_*Z*4_ = < (0.6632, 0.6963), (0.1333, 0.7000), (0.1695, 0.8206)>	*Y*(*s*_*Z*1_) = 0.7317, *Y*(*s*_*Z*2_) = 0.7440, *Y*(*s*_*Z*3_) = 0.6762, *Y*(*s*_*Z*4_) = 0.7431	*Q*_2_ > *Q*_4_ > *Q*_1_ > *Q*_3_
MDM method based on the SVNWAA operator [33]	*s*_1_ = < 0.6701, 0.1248, 0.2000 > , *s*_2_ = < 0.7451, 0.1558, 0.2000 > , *s*_3_ = < 0.6701, 0.2277, 0.2606 > , *s*_4_ = < 0.6682, 0.1248, 0.1421>	*Y*(*s*_1_) = 0.7818, *Y*(*s*_2_) = 0.7964, *Y*(*s*_3_) = 0.7273, *Y*(*s*_4_) = 0.8004	*Q*_4_ > *Q*_2_ > *Q*_1_ > *Q*_3_
MDM method based on the SVNWGA operator [33]	*s*_1_ = < 0.6653, 0.1333, 0.2000 > , *s*_2_ = < 0.7234, 0.2095, 0.2000 > , *s*_3_ = < 0.665, 0.2335, 0.3642 > , *s*_4_ = < 0.6632, 0.1333, 0.1695>	*Y*(*s*_1_) = 0.7773, *Y*(*s*_2_) = 0.7713, *Y*(*s*_3_) = 0.6892, *Y*(*s*_4_) = 0.7868	*Q*_4_ > *Q*_1_ > *Q*_2_ > *Q*_3_

By comparing the developed MDM approach with existing neutrosophic MDM method [33] in Table 1, we see that there is the ranking difference between them. The developed MDM approach based on the NZNWAA or NZNWGA operator indicates that the ranking is *Q*_2_ > *Q*_4_ > *Q*_1_ > *Q*_3_ and the best alternative is *Q*_2_, while the MDM method based on the NZNWAA or SVNWGA operator [33] indicates that the ranking is *Q*_4_ > *Q*_2_ > *Q*_1_ > *Q*_3_ or *Q*_4_ > *Q*_1_ > *Q*_2_ > *Q*_3_ and the best alternative is *Q*_4_.

### Discussion

From the decision results of Table 1, it is obvious that the MDM methods with different decision information can affect the ranking orders. The reason resulting in the different ranking is that the developed MDM approach uses the hybrid assessment information of both neutrosophic values and neutrosophic measures of corresponding reliabilities, while existing neutrosophic MDM method [33] only uses the assessment information of single-valued neutrosophic values without considering the related reliability measures. Clearly, the introduced reliability measures not only can enhance the information expression and credibility of the evaluation results but also can impact on the ranking order of alternatives, which show the effectiveness and rationality of the developed MDM approach. Since NZNs indicate more ability to depict the human knowledge and judgments by neutrosophic values and the reliability measures related to the neutrosophic values, NZNs richen the measure information of reliability related to the neutrosophic values in indeterminate and inconsistent setting. Hence, the information expression of NZN is superior to that of the single neutrosophic value or the single Z-number in MDM problems. Then, the developed MDM approach in this paper indicates more generalized form to extend existing neutrosophic MDM theory and methods because the existing neutrosophic MDM method is only the special case of this study and cannot carry out the MDM problem with NZN information. Therefore, the developed MDM approach can overcome the flaw of existing neutrosophic MDM theory and methods and strengthen the MDM reliability and effectiveness, which show the highlighting advantages under the environment of NZNs.

## Conclusion

In the original study, the proposed NZN set can solve the hybrid information expression problem of both neutrosophic values and corresponding reliability measures and overcome the flaw of missing reliability measures in existing neutrosophic set. Then, the proposed basic operations, score function, and NZNWAA and NZNWGA operators of NZNs provided effective and reasonable mathematical tools for the information aggregation of NZNs and MDM modeling in the setting of NZNs. Further, the developed MDM approach using the NZNWAA and NZNWGA operators and the score function can solve MDM problems with NZN information as the extension of existing neutrosophic MDM methods. The decision results of the illustrative example about the selection problem of business partners indicated the applicability and effectiveness of the developed MDM approach in NZN setting.

However, the developed MDM approach extends existing neutrosophic MDM theory and methods and provides a new way for solving MDM problems with NZNs. Since as the first time study, the ranking method based on the score function and the NZNWAA and NZNWGA operators are the most basic algorithms for MDM problems with NZNs, we should continue to propose the new aggregation operators (e.g., Bonferroni mean, Heronian mean, Dombi aggregation operators) and ranking methods of NZNs for improving neutrosophic MDM methods and to extend them to the applications of group decision making, medical diagnosis, pattern recognition, optimization programming etc.
